# The complete mitochondrial genome of *Liparis tessellatus* and its phylogenetic analysis

**DOI:** 10.1080/23802359.2020.1768933

**Published:** 2020-05-28

**Authors:** Ju-Hyung Jeon, Jeong-Nam Yu, Hyung-Joo Jin, Deuk-Hee Jin

**Affiliations:** a Department of Marine Molecular Bioscience, Gangneung-Wonju National University, Gangneung, Korea; b Biodiversity Conservation and Change Division, Freshwater Biodiversity Research Bureau, Nakdonggang National Institute of Biological Resource (NNIBR), Sangju-Si, Korea

**Keywords:** *Liparis tessellatus*, mitochondrial genome, phylogenetic analysis

## Abstract

*Liparis tessellatus* is a cubed snailfish that inhabits the northwestern region of the Pacific Ocean. The family Liparidae is difficult to distinguish morphologically due to the typical body color and shape variation, which are used interchangeably due to the differences in local dialects. Therefore, we determined the complete mitochondrial genome sequence of *L. tesellatus*. The mitochondrial genome length of *L. tesellatus* was determined as 17,221 bp, which consisted of 22 tRNA genes, two rRNA genes, 13 protein-coding genes (PCGs), and a control region (D-loop). The base composition was as follows: 28.6% of A, 29.5% of T, 26.5% of C, and 15.4% of G. The phylogenetic analysis revealed that *L. tesellatus* clustered together with different species of the genus Liparis. Thus, the complete mitochondrial genome sequence provided herein would further help in understanding the evolution of *Liparis* species.

*Liparis tessellatus* is a cube snailfish that belongs to the family Liparidae and is found to inhabit the northwestern region of the Pacific Ocean. There are 10 species from three genera that are reported to be present in Korean waters (Ji et al. 2012; Park et al. 2013). The family Liparidae is difficult to distinguish morphologically due to the typical body color and shape variation, which is used interchangeably due to the differences in local dialects (Song et al. 2018). They are morphologically similar to the larvae, as both of them share a common etymology. In Korea, *L. ochotensis* and *L. tanakae* are found to be morphologically similar, and *L. tanakae* and *L. tessellatus* are used interchangeably in nomenclature. Therefore, it was important to determine the mitochondrial genome of *L. tessellatus*.

A voucher specimen was collected from Uljin, Gyeongsangbuk Province, Korea (N 37°05’50.2”, E 129°52’29.4”) and stored at the Laboratory of Marine Molecular Biology, Gangneung-Wonju National University, Korea (sample collection ID: GWNU-P-MMB1300006, PKU 58483). Total genomic DNA was extracted from the muscle tissue of the specimen using the DNeasy Blood and Tissue Kit (Qiagen GmbH, Hilden, Germany). PCR products were sequenced through the primer walking method using the ABI 3730XL DNA Analyzer (Applied Biosystems Inc., Foster City, CA, USA). The sequences were assembled, aligned, and annotated using MEGA6.0 (Tamura et al. 2013) and tRNAscan-SE 2.0 (Chan and Lowe 2019). The genome sequence of *L. tessellatus* along with its gene annotations was deposited in GenBank under the accession number MN880630.

The complete mitochondrial genome of *L. tessellatus* was determined to be 17,221 bp in length, which consisted of 22 tRNA genes, two rRNA genes, 13 protein-coding genes (PCGs), and a control region (D-loop). This is similar to what was previously reported for other vertebrates (Inoue et al. 2000). The base composition was as follows: 28.6% of A, 29.5% of T, 26.5% of C, and 15.4% of G. The overall A + T and C + T contents were 58% and 42%, respectively. Of the 37 genes, nine genes were located on the light strand (L-strand), and one protein-coding gene (NADH dehydrogenase subunit 6 (*ND6*)), eight tRNAs (Gln, Tyr, Ala, Asn, Cys, Ser, Glu, and Pro), and the remaining genes were located on the heavy strand (H-strand). Of the 13 protein-coding genes, 12 genes started with the codon ATG and only the Cytochorome c Oxidase 1 (*CO1*) gene started with the codon GTG (Hwang et al. 2012). Moreover, all the tRNAs ranged between 66 and 74 bp, exhibiting typical clover-leaf secondary structure. A control region (D-loop) of 1202 bp was located between the tRNA-Phe and -Pro.

Furthermore, the neighbour-joining (NJ) gene tree was constructed with MEGA using the *CO1* partial gene sequence and was compared with the gene tree of our previous study (Sim et al. 2020) (Figure 1). To determine the phylogenetic position of *L. tessellatus*, the *CO1* sequences of nine species obtained from GenBank and one sequence from the present study were used. Phylogenetic analysis revealed that *L. tesellatus* clustered together with the species of the genus Liparis. Thus, the genetic information of *L. tessellatus* deduced in this study will contribute to the identification and better understanding of the evolutionary flexibility of diverse marine organisms, in addition to the family Liparidae.

**Figure 1.**
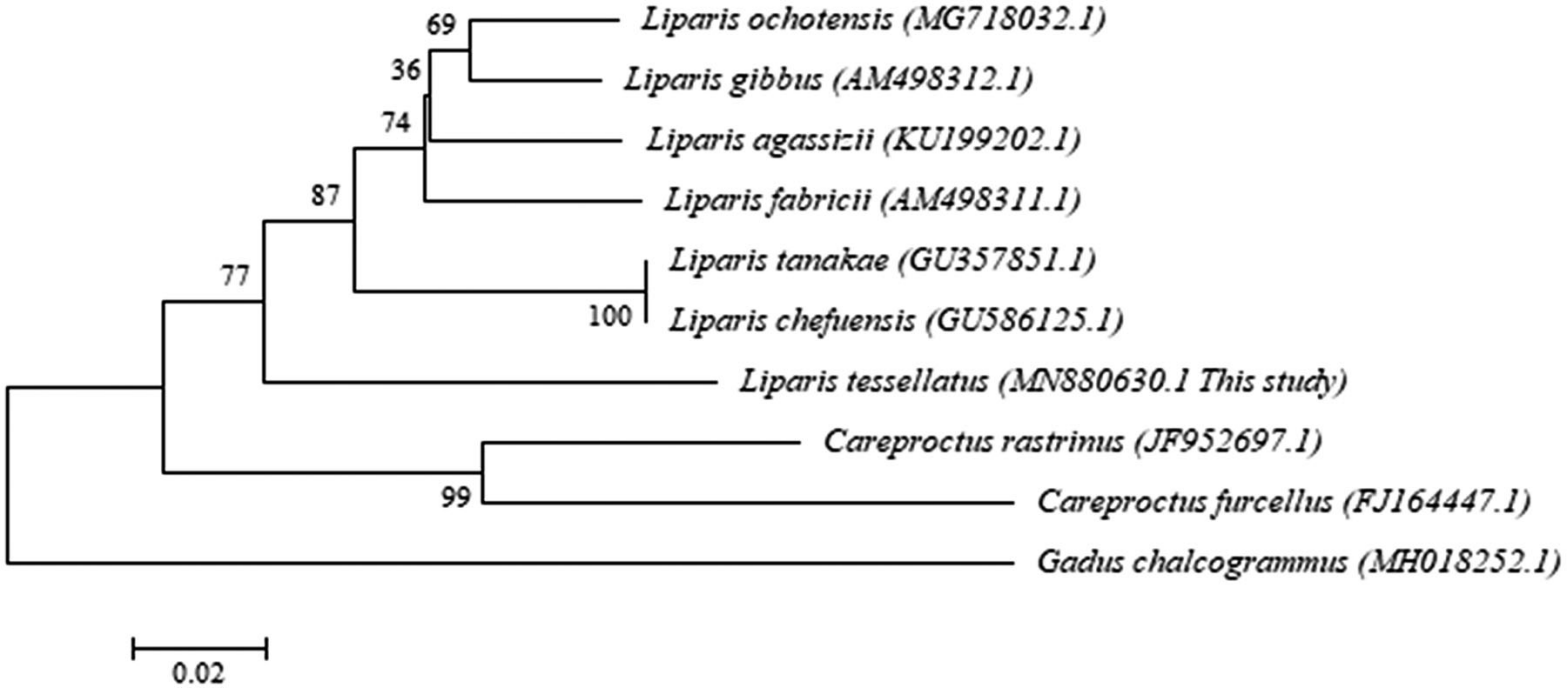
The neighbour-joining (NJ) gene tree was constructed with MEGA using the CO1 partial gene sequence, which was compared with the gene tree of our previous study (Sim et al. 2020). The gene tree of *Liparis tessellatus* along with other Liparidae species was generated based on the CO1 partial gene sequences derived from the mitochondrial genome using the NJ analysis. Bootstrap replicates were conducted 10,000 times.

## Data Availability

The data that support the findings of this study are openly available in GenBank at https://www.ncbi.nlm.nih.gov/, accession number MN880630.
